# Residential Radon and Histological Types of Lung Cancer: A Meta-Analysis of Case‒Control Studies

**DOI:** 10.3390/ijerph17041457

**Published:** 2020-02-24

**Authors:** Cong Li, Chunhong Wang, Jun Yu, Yongsheng Fan, Duanya Liu, Wenshan Zhou, Tingming Shi

**Affiliations:** 1 Hubei Provincial Center for Disease Control and Prevention, 6 Zhuodaoquan North Road, Wuhan 430079, Hubei, China; congli@whu.edu.cn; 2 Department of Preventive Medicine, School of Health Sciences, Wuhan University, Donghu Road 115, Wuhan 430071, Hubei, China; wchunhong027@whu.edu.cn (C.W.); junyu6699@whu.edu.cn (J.Y.); fanyongsheng1993@163.com (Y.F.); ldy0330@whu.edu.cn (D.L.)

**Keywords:** residential radon, lung cancer, histology, meta-analysis, case‒control study

## Abstract

Epidemiological studies on residential radon exposure and the risk of histological types of lung cancer have yielded inconsistent results. We conducted a meta-analysis on this topic and updated previous related meta-analyses. We searched the databases of Cochrane Library, Embase, PubMed, Web of Science and Chinese National Knowledge Infrastructure for papers published up to 13 November 2018. The pooled odds ratio (OR) and 95% confidence interval (CI) were calculated using fixed and random effects models. Subgroup and dose‒response analyses were also conducted. This study was registered with PROSPERO (No. CRD42019127761). A total of 28 studies, which included 13,748 lung cancer cases and 23,112 controls, were used for this meta-analysis. The pooled OR indicated that the highest residential radon exposure was significantly associated with an increased risk of lung cancer (OR = 1.48, 95% CI = 1.26–1.73). All histological types of lung cancer were associated with residential radon. Strongest association with small-cell lung carcinoma (OR = 2.03, 95% CI = 1.52–2.71) was found, followed by adenocarcinoma (OR = 1.58, 95% CI = 1.31–1.91), other histological types (OR = 1.54, 95% CI = 1.11–2.15) and squamous cell carcinoma (OR = 1.43, 95% CI = 1.18–1.74). With increasing residential radon levels per 100 Bq/m^3^, the risk of lung cancer, small-cell lung carcinoma and adenocarcinoma increased by 11%, 19% and 13%, respectively. This meta-analysis provides new evidence for a potential relationship between residential radon and all histological types of lung cancer.

## 1. Introduction

Lung cancer is the most commonly diagnosed cancer in the world and the leading cause of death from cancer [1]. According to GLOBOCAN (2018), lung cancer accounted for 11.6% (2.1 million) of total cancer cases and 18.4% (1.8 million) of cancer deaths in 2018 [2]. This malignancy has a diverse histological structure. Lung cancer is generally divided into two main histological groups, namely, small-cell lung carcinoma (SCLC, approximately 15% of all lung cancers) and non-small-cell lung cancer (NSCLC, approximately 85% of all lung cancers). NSCLC can be divided into three predominant histological subtypes, namely, adenocarcinoma, squamous cell carcinoma and large-cell carcinoma [3,4,5]. SCLC is a highly aggressive tumour. Contrary to NSCLC, SCLC is characterised by rapid doubling time, high growth fraction, early metastatic spread and initial responsiveness to chemotherapy and radiation [6]. Nevertheless, despite promising initial responses to treatment, nearly all patients with lung cancer had a relapse in the first two years, with a poor prognosis. The systematic treatment of SCLC has seen little progress in the past three decades. Therefore, the five-year survival rate is less than 7%, and most patients only survive for one year or less after diagnosis [7,8]. By contrast, significant progress has been achieved over the past 50 years in the treatment of the most common type of lung cancer, namely, NSCLC, with a five-year survival rate of 15% [9,10].

Radon is a naturally occurring radioactive noble gas that arises in the radioactive decay chain of uranium-238. This gas is the main source of natural radiation for human beings and widely exists in the daily environment, such as homes, workplaces and schools [11]. The main sources of indoor radon are soil around the foundation, building materials, fuels and domestic water. Residential radon concentration depends not only on housing factors (such as the type of housing, decoration materials, floor and age of housing), but also on environmental conditions (such as temperature, humidity and atmospheric pressure), time factors (such as season or day versus night) and the ventilation capacity of indoor and outdoor air [12]. In a radon-prone area in Spain, the residential radon concentration of new dwellings is higher than that of traditional dwellings, and renovated traditional dwellings have higher residential radon than nonrenovated ones [13]. A study in South Korea shows that the effects on radon concentration in the residential environment are in the order of location, construction year and season [14].

Radon is one of the 19 environmental carcinogens recognised by the World Health Organization (WHO). The International Agency for Research on Cancer includes radon and its progeny as carcinogenic factors. According to the U.S. Environmental Protection Agency (EPA), radon has been identified as the second leading cause of lung cancer, after cigarette smoking and the first risk factor for nonsmokers [15]. Radon causes an estimated 3%–14% of lung cancer, depending on the average level of radon in the country and the smoking prevalence [16]. Studies on radon as a risk factor for lung cancer have initially focused on uranium miners exposed to high radon levels. Then, residential radon exposure of the general population has been attracting increasing attention. In November 2014, the International Committee on Radiological Protection (ICRP) issued a special report entitled “Radiological Protection against Radon Exposure” (ICRP 126), emphasising that public exposure to indoor radon should not be neglected. In addition, residential radon exposure is particularly relevant in stomach and brain cancer [17].

Two meta-analyses [18,19] and three pooled analyses [20,21,22] of case–control studies have suggested that residential radon exposure may be associated with an increased risk of lung cancer, and another meta-analysis [23] yielded a contrasting conclusion. Histological classification of lung cancer is important for the treatment and prognosis of patients. However, the effect of residential radon exposure on the risk of the histological types of lung cancer has yet to be considered in the aforementioned meta-analyses. The relationship between residential radon exposure and the risk of the different histological types of lung cancer has become the focus of recent attention. A study [24] shows that SCLC is apparently the histological type of lung cancer most closely related to residential radon. Nevertheless, this study is only a systematic review without a quantitative analysis. To the best of our knowledge, no meta-analyses have quantitatively assessed whether a relationship exists between residential radon exposure and the risk of the different histological types of lung cancer.

Therefore, we conducted a meta-analysis of case‒control studies for the general population to quantitatively assess the strength of the relationship between residential radon and the major histological types of lung cancer. We also updated the previous related meta-analyses. We believe that a new combined analysis is necessary and beneficial.

## 2. Materials and Methods 

The meta-analysis in this study was reported according to the Preferred Reporting Items for Systematic Reviews and Meta-Analyses Statement [25] and was registered with the PROSPERO International Prospective Register of Systematic Reviews (No. CRD42019127761).

### 2.1. Search Strategy and Selection Criteria

We searched the databases of Cochrane Library, Embase, PubMed, Web of Science and Chinese National Knowledge Infrastructure (CNKI) to identify all case‒control studies related to residential radon and lung cancer published up to 13 November 2018. As detailed in Supplementary Table S1, no date restrictions were imposed, and search terms included “radon,” “residential radon,” “lung neoplasm,” “lung cancer” and “lung tumour.” Language was not restricted in the original search; however, all identified studies were in English and Chinese. Moreover, a manual search using reference lists of previous relevant published systematic reviews and meta-analysis was performed.

We identified and defined the selection criteria. Two independent reviewers (C.L. and W.Z.) screened all titles and abstracts for inclusion and the full-text articles of all case‒control studies identified. Conflicts were resolved by consensus within the review team. The inclusion criteria were as follows. (a) The studies had a case‒control design with a hospital- or population-based design. (b) The exposure of interest was residential radon, which was determined by a radon detector by measurements over at least three months. (c) The outcome of interest was histologically confirmed lung cancer. (d) The subjects performed only in the general population. (e) Finally, odds ratios (ORs)/excess ORs (EORs) with their corresponding 95% confidence intervals (CIs) were reported (or sufficient data were provided to calculate these values).

### 2.2. Data Extraction

Two independent review authors (C.L. and J.Y.) extracted and recorded data from the included studies. All discrepancies were discussed to reach a consensus. A predefined form was then used to extract information from each study as follows: first author’s surname; year of publication; country of study; participant characteristics, including age range, sex and smoking situation; sample size (controls and cases); sources of controls (categorised as population- or hospital-based studies); histological types of lung cancer; radon dosimetry, including detector type, duration of measurements and place of measurements; and ORs, EORs with their corresponding 95% CIs and statistical adjustment for the main confounding factors of interest. The qualities of the included studies were assessed independently by two authors (Y.F. and D.L.) using the Newcastle–Ottawa quality assessment scale, which ranged from 0 to 9; we defined high-quality studies as scoring 6–9 points [26].

### 2.3. Statistical Analysis

The primary outcomes of the meta-analysis were the ORs, EORs and their corresponding 95% CIs for lung cancer in patients with the highest radon exposure compared with the non/lowest radon-exposed population. Study-specific ORs, EORs and corresponding 95% CIs for highest versus non/lowest radon exposure levels were extracted; and log ORs and log EORs were then weighted by the inverse of their variances to obtain a pooled OR, EOR and their 95% CIs. Initially, all studies were pooled together, and a sensitivity analysis was performed to assess the influences of the methodological concerns by grouping them into different subgroups, including histological subtype, study population, study design, smoking status, sex and duration of radon measurements. In addition, we performed dose‒response analyses of radon on lung cancer and its histological types. Q tests and I^2^ statistics were used to evaluate the statistical heterogeneity among studies. We used fixed effects models in the absence of significant heterogeneity on the Q tests (*p* > 0.1) and I^2^ statistic value ≤50%; otherwise, we used random effects models. A funnel plot and Begg’s test were applied to assess potential publication bias [27,28]. We used Stata (version 13.0; StataCorp, College Station, TX, USA) to analyse the data, and the level of significance was considered at 5%.

## 3. Results

### 3.1. Study Selection

**Figure 1:**
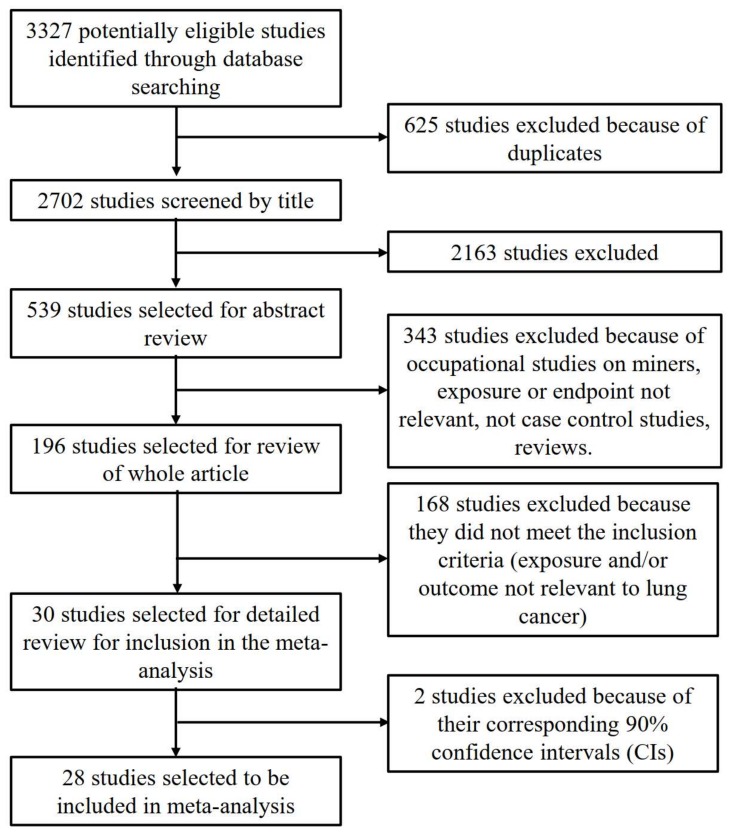
Flowchart of the study selection process.

We retrieved 6117 citations (eight, 568, 659, 1758 and 334 from Cochrane Library, Embase, PubMed, Web of Science and CNKI, respectively), after removing 625 duplicates. A total of 196 were selected for full text review. The process of study selection and exclusion is shown in Figure 1. After assessing the full text of the relevant articles, 30 studies were selected for detailed review for inclusion in the meta-analysis. From the 30 studies, two reports [29,30] were excluded because their corresponding CIs were 90%, not 95%. Finally, 28 studies [31,32,33,34,35,36,37,38,39,40,41,42,43,44,45,46,47,48,49,50,51,52,53,54,55,56,57,58], which included 13,748 lung cancer cases and 23,112 controls, were used for the meta-analysis to assess residential radon exposure and the risk of lung cancer. Among the 28 studies, 20 studies discussed the histological subtypes of lung cancer. However, two [37,56] of them did not meet the inclusion criteria because ORs/EORs with their corresponding 95% CIs were not reported. Finally, 18 studies have been considered for analyses related to histological types. Hence, 12 studies were conducted for histology subgroup analyses and eight studies for dose‒response analyses.

### 3.2. Study Characteristics

The main characteristics of the 28 studies are summarised in Table 1. These studies were published between 1989 and 2015. The sample sizes ranged from 28 to 3185. A total of eight studies were hospital-based, 18 studies were population-based and two studies were hospital- or population-based. In addition, 18 studies were conducted in Europe, seven studies in North America and three studies in Asian regions. In terms of environmental conditions, 12 studies were conducted in radon-prone areas. The type of housing was illustrated in four studies [31,33,43,47]. Two studies [31,33] presented building materials, whilst one [32] study showed the building’s floor and age. For radon dosimetry, one study [31] used the thermoluminescence dosimeter designed by the National Institute of Radiation Protection in Sweden, whilst others used alpha track detectors. The detectors were usually placed in the participants’ bedroom or living room. Radon was measured for one year in 16 studies, compared with less than one year in other studies. We also evaluated the quality of the 28 studies, all of which had a score of 6 or higher, as shown in Table 1. Thus, all 28 studies were of high quality.

### 3.3. Overall Pooled Analysis

**Figure 2:**
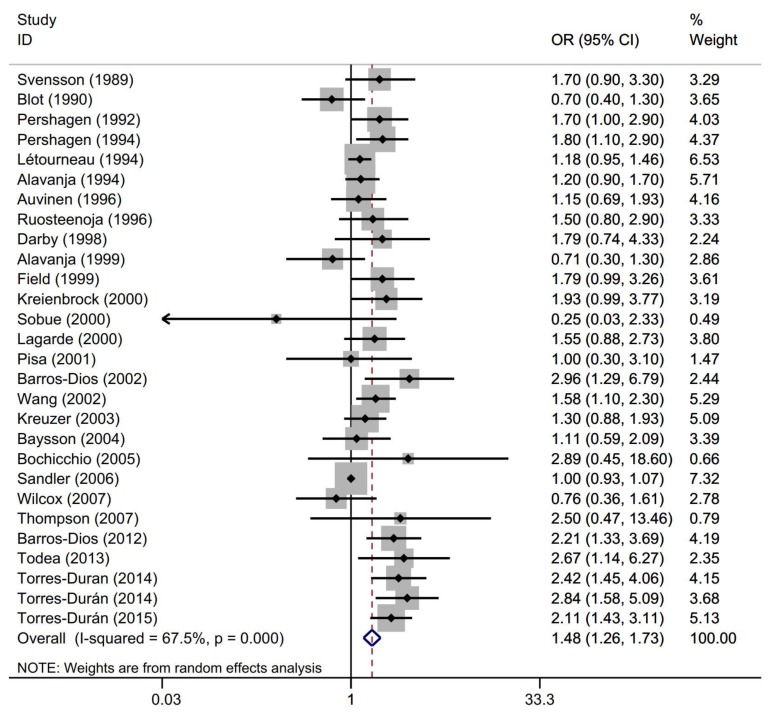
Forest plot for the pooled estimates of the risk of lung cancer with residential radon exposure (highest versus non/lowest).

The multivariable adjusted ORs of lung cancer for the highest and non/lowest residential radon exposures are shown in Figure 2 and Table 2. A random effects model was used to calculate the pooled estimated OR because substantial heterogeneity was found across these studies (I^2^ = 67.5%, *p*-heterogeneity = 0.000). The pooled OR indicated a statistically significant association between the highest residential radon exposure and an increased risk of lung cancer (OR = 1.48, 95% CI = 1.26–1.73).

In the sensitivity analysis, removing one study at a time did not significantly affect the pooled OR, ranging from 1.52 (95% CI = 1.29–1.77) to 1.44 (95% CI = 1.23–1.68). However, the heterogeneity was primarily due to one study by Sandler et al. (2006). After excluding the study, the heterogeneity significantly declined (I^2^ = 43.1%, *p*-heterogeneity = 0.010), whereas the summary estimate was substantially unchanged (OR = 1.52, 95% CI = 1.31–1.76). For the overall pooled analysis, no significant evidence of publication bias, as shown by the funnel plot (Figure S1) and Begg’s test (Z = 0.73, *p* = 0.465). 

### 3.4. Histology Subgroup Analyses

**Figure 3:**
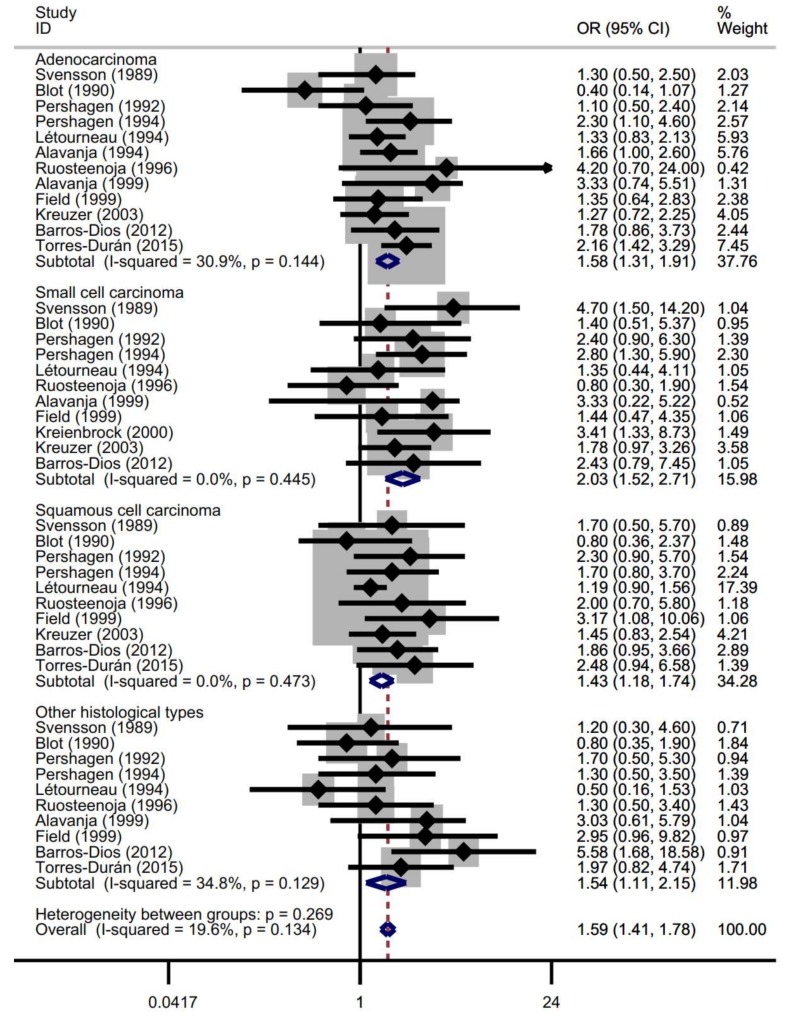
Forest plot of the associations between lung cancer risk and residential radon exposure (highest versus non/lowest) stratified by types of histology.

Table 2 and Figure 3 show the results of the histology subgroup analyses, in which fixed effects models were used due to insignificant heterogeneity. Statistically significant associations were observed in all histological subtypes of lung cancer, in which 12 studies [31,32,33,34,35,36,38,40,41,48,54,58] reported on the associations among adenocarcinoma (OR = 1.58, 95% CI = 1.31–1.91), 11 studies [31,32,33,34,35,38,40,41,42,48,54] on the associations among SCLC (OR = 2.03, 95% CI = 1.52–2.71), 10 studies [31,32,33,34,35,38,41,48,54,58] on the associations among squamous-cell carcinoma (OR = 1.43, 95% CI = 1.18–1.74) and 10 studies [31,32,33,34,35,38,40,41,54,58] on associations among other histological types (OR = 1.54, 95% CI = 1.11–2.15). For the histology subgroup analyses, no evidence of publication bias was found, as suggested by the funnel plot (Figure S2) and Begg’s test (Z = 1.59, *p* = 0.112).

### 3.5. Other Subgroup Analyses

Table 2 presents the results of other subgroup analyses stratified by study population, study design, smoking status, sex and duration of radon measurements. The subgroup analyses were performed using the random effects models. In the subgroup analysis by study population, a significantly positive association of residential radon exposure with lung cancer risk was observed in the studies conducted in Europe (OR = 1.77, 95% CI = 1.54–2.03,) but not in North America (OR = 1.09, 95% CI = 0.94–1.27) or Asia (OR = 0.93, 95% CI = 0.42–2.06). When stratified by study design, smoking status, sex and duration of radon measurements, a statistically significant association was found for all stratified analyses, except for sex. Heterogeneity was significantly reduced among studies conducted in Europe, hospital-based controls, hospital and population-based controls and duration of radon measurements of less than 12 months.

### 3.6. Dose‒Response Analyses

We performed dose‒response analyses of residential radon exposure on the risk of lung cancer and histological types in nine studies (Table 3). Fixed effects models were used due to insignificant heterogeneity (overall: I^2^ = 0.0%, *p*-heterogeneity = 0.994; adenocarcinoma: I^2^ = 0.0%, *p*-heterogeneity = 0.604; SCLC: I^2^ = 0.0%, *p*-heterogeneity = 0.983; squamous cell carcinoma: I^2^ = 0.0%, *p*-heterogeneity = 0.490; and other histological types: I^2^ = 52.4%, *p*-heterogeneity = 0.122). All studies showed a positive dose‒response relationship between residential radon exposure and the risk of lung cancer. With the increase in residential radon exposure per 100 Bq/m^3^, the risk of lung cancer increased from 5% to 28%. The pooled EOR also indicated that the risk of lung cancer increased significantly by 11% with increasing residential radon levels per 100 Bq/m^3^ (EOR = 0.11, 95% CI = 0.05–0.17). Among the histological types, residential radon showed a significant dose‒response relationship with SCLC (EOR = 0.19, 95% CI = 0.07–0.32), followed by adenocarcinoma (EOR = 0.13, 95% CI = 0.01–0.25). However, no dose‒response relationship was found in squamous cell carcinoma (EOR = 0.00, 95% CI = −0.12–0.12) and other histological types (EOR = −0.03, 95% CI = −0.22–0.16).

## 4. Discussion

In this meta-analysis of 28 case‒control studies, residential radon exposure was significantly associated with the risk of lung cancer and its histological types. To the best of our knowledge, this meta-analysis is the first to quantitatively assess the strength of the relationship between residential radon and the major histological types of lung cancer. The most remarkable finding is that all histological types of lung cancer were associated with residential radon, with varying strengths of association among the major types. Previous pooled analysis [20] of European also confirmed the finding. SCLC had the strongest relationship with residential radon followed by adenocarcinoma. This result was consistent with those from the results of dose‒response analyses. With increasing residential radon levels per 100 Bq/m^3^, the risks of SCLC and adenocarcinoma increased by 19% and 13%, respectively. However, no significant association was found between residential radon and squamous cell carcinoma and other histological types. A previous pooled analysis [21] of North American studies has shown that there is a dose‒response relationship between residential radon and squamous cell carcinoma and other histological types. This result was possibly due to the small number of studies included in the dose‒response analyses, leading to unreliable results. 

A meta-analysis [59] showed that all histological types of lung cancer were significantly associated with cigarette smoking. Strongest association was found with SCLC, followed by squamous cell carcinoma, large-cell carcinoma and adenocarcinoma. These results are similar to our meta-analysis results. Smoking and radon were associated with all histological types of lung cancer, and the strongest association was found with SCLC. Several differences were also observed. Adenocarcinoma was the second histological type associated with radon, but was the weakest associated with smoking, contrary to the results for squamous cell carcinoma.

Although radon and smoking have their own pathogenic mechanisms and characteristics as two independent lung cancer risk factors, increasing evidence shows that they are closely related. The occurrence and development of lung cancer is a complex biological process. In this process, many common mechanisms occur between radon and smoking, from the early stage of injury to all stages of carcinogenesis [60]. Therefore, radon and smoking may have a synergistic effect on the occurrence and development of lung cancer [61]. In a study on nonsmokers, the cancer risk is apparent for levels of 200 Bq/m^3^ [56]. However, the U.S. EPA and WHO recommended action levels of 148 Bq/m^3^ and 100 Bq/m^3^, respectively [16,62]. These recommendations are based on studies that mainly involved smokers. In this meta-analysis, the risk of lung cancer increased with smoking under the highest radon exposure. The risk of lung cancer in smokers with the highest exposure to radon is 14.8 times higher than in smokers with no/lowest exposure to radon.

When the subgroup analysis was conducted by study population, a statistically significant positive association was only observed in Europe, but not in North America and Asia. This phenomenon may be due to the different levels of radon concentration in each region and the insufficient number of studies included. Although 58.5% of lung cancer cases occurred in Asia in 2018 [2], few studies on lung cancer and radon were conducted in Asia, and only three studies were included in this meta-analysis. In the stratified analysis by sex, the pooled OR in females was lower than males and females. This may be because of the greater number of male smokers exposed to radon than females. The mortality rate of male lung cancer in 2018 was also significantly higher than that of females [2]. Radon monitoring equipment could not affect the comparison of the results; however, the radon measurement time affected the comparison of results. The higher the risk of lung cancer was for householders with a measurement period of less than one year. This may be because short-term measurements of radon concentrations are inaccurate, exaggerating the effect of radon on lung cancer. Only five studies presented the environmental conditions of the study subjects, and one [47] of these studies provided the OR values for the type of housing. The type of housing was underground, and the risk of lung cancer exposure to high radon was 2.03 times higher than that to low radon. No difference was found for the standard housing type.

Our meta-analysis has several advantages. First, ours is the first to focus exclusively on the effect of residential radon on the histological types of lung cancer. We also updated the previous related meta-analyses. Second, we searched five Chinese and English databases and conducted manual searches to ensure that relevant research would be included in this meta-analysis. Finally, insignificant heterogeneity was found in the histology subgroup and dose‒response analyses. Our meta-analysis also has several limitations. First, only case‒control studies were included. A case‒control study is a retrospective observational study that is prone to various biases. On the basis of the literature search results, some cohort studies have been conducted on radon occupational exposure; however, no cohort study has been conducted on radon exposure in the general population. Second, our results may be affected by the classification of radon exposure. Given the distinct radon concentrations in different regions, the radon exposure classifications designed for each study also vary. We can only extract the OR values of the highest versus non/lowest radon-exposed groups in each study. Third, publication bias may have occurred because only published studies were included in our meta-analysis.

## 5. Conclusions

This meta-analysis of 28 case‒control studies, which included 13,748 lung cancer cases and 23,112 controls, suggests that residential radon is a risk factor in all histological types of all lung cancer. Stronger associations were found with SCLC and adenocarcinoma than with squamous cell carcinoma and other histological types. Residential radon exposure remains a major concern worldwide, and appropriate measures should be undertaken to reduce radon exposure to ensure the health of environmental conditions and residents.

## Figures and Tables

**Table 1:** Characteristics of studies included in the meta-analysis.

Study	Study Location	Study Design	Sex	Age (Years)	Smoking Status	Cases/Controls (*n*)	Exposure Comparison (Bq/m^3^)	OR (95% CI)	Adjusted Covariates	Quality Score
Svensson et al. (1989) ^a, b^ [31]	Sweden	PCC, HCC	F	All	mixed	210/400	<4500 vs. ≥6000	1.7(0.9–3.3)	age, smoking, and municipality of residency	6
Blot et al. (1990) ^a^ [32]	China	PCC	F	30–69	mixed	308/356	<70 vs. ≥296	0.7 (0.4–1.3)	age, education, smoking status, and an index of indoor air pollution	8
Pershagen et al. (1992) ^a^ [33]	Sweden	PCC, HCC	F	All	mixed	210/209	≤75 vs. ≥151	1.7 (1.0–2.9)	age, smoking, and municipality of residency	7
Pershagen et al. (1994) ^a, b^ [34]	Sweden	PCC	M/F	35–74	mixed	1360/2847	≤50 vs. >400	1.8 (1.1–2.9)	Age, sex, smoking, occupation, area of residence	7
Letourneau et al. (1994) ^a^ [35]	Canada	PCC	M/F	35–80	mixed	738/738	<25 vs. ≥200	1.18(0.95–1.46)	smoking and education	7
Alavanja et al. (1994) ^a^ [36]	USA	PCC	F	30–84	NS	538/1138	<30 vs. 91–566	1.2 (0.9–1.7)	Age and smoking	8
Auvinen et al. (1996) [37]	Finland	PCC	M/F	All	mixed	517/517	≤49 vs. 400–1277	1.15 (0.69–1.93)	smoking	7
Ruosteenoja et al. (1996) ^a, b^ [38]	Finland	PCC	M	0–64	mixed	164/331	≤95 vs. >186	1.5 (0.8–2.9)	age, smoking intensity, and quitting of smoking prior to 1979	8
Darby et al. (1998) [39]	UK	HCC	M/F	<75	mixed	982/3185	<25 vs. ≥400	1.79 (0.74–4.33)	age, sex, smoking, county of residence and social class	7
Alavanja et al. (1999) ^a^ [40]	USA	PCC	F	30-84	mixed	247/299	<37 vs. ≥148	0.71 (0.3–1.3)	Age, education, smoking, previous lung disease, and vegetable consumption	8
Field et al. (2000) ^a^ [41]	USA	PCC	F	40–84	mixed	413/614	≤57 vs. >228	1.79 (0.99–3.26)	Age, smoking, and education	8
Kreienbrock et al. (2000) ^a, b^ [42]	Germany	PCC	M/F	≤75	mixed	1449/2297	<50 vs. >140	1.93 (0.99–3.77)	smoking and asbestos	8
Sobue et al. (2000) [43]	Japan	PCC	M/F	≥40	mixed	28/36	≤24 vs. ≥100	0.25 (0.03–.33)	sex, year of birth, smoking status and occupational history	7
Lagarde et al. (2001) [44]	Sweden	PCC	M/F	≥28	NS	258/487	<50 vs. >140	1.55 (0.88–2.73)	Age, sex, passive smoking, area of current residence, and socioeconomic status	8
Pisa et al. (2001) ^b^ [45]	Italy	PCC	M/F	All	mixed	138/291	<40 vs. ≥200	1.0 (0.3-3.1)	Age, sex, and smoking	7
Barros-Dios et al. (2002) [46]	Spain	PCC	M/F	≥35	mixed	163/241	<36.9 vs. ≥148	2.96 (1.29–6.79)	Age, sex, and family history	8
Wang et al. (2002) ^b^ [47]	China	PCC	M/F	30-75	mixed	768/1659	<100 vs. ≥300	1.58 (1.1–2.3)	Age, sex, prefecture, smoking and socioeconomic factors	8
Kreuzer et al. (2003) ^a^ [48]	Germany	PCC	M/F	<76	mixed	1192/1640	<50 vs. >140	1.30 (0.88–1.93)	Smoking, occupational asbestos	8
Baysson et al. (2004) ^b^ [49]	France	HCC	M/F	<75	mixed	486/984	<50 vs. >400	1.11 (0.59–2.09)	Age, sex, region, smoking and occupational exposure to asbestos and carcinogens	7
Bochicchio et al. (2005) ^b^ [50]	Italy	HCC	M/F	35–90	mixed	384/401	<50 vs. >400	2.89 (0.45–18.6)	sex, age, sex X age, area of residence in Lazio, smoking and dietary variables	7
Sandler et al. (2006) [51]	USA	PCC	M/F	40–79	mixed	1474/1811	<18 vs. ≥53	1.00 (0.93–1.07)	Age, sex, and smoking	7
Wilcox et al. (2008) ^b^ [52]	USA	PCC	M/F	All	mixed	561/740	<25 vs. ≥150	0.76(0.36–1.61)	Age, sex, and smoking	8
Thompson et al. (2008) [53]	USA	HCC	M/F	>40	mixed	200/397	<25 vs. ≥250	2.50 (0.47–13.46)	smoking, residency, job exposure, income, and education	7
Barros-Dios et al. (2012) ^a, b^ [54]	Spain	HCC	M/F	>30	mixed	308/484	<50 vs. >147	2.21 (1.33–3.69)	age, sex, and tobacco consumption	7
Todea et al. (2013) [55]	Romania	PCC	M/F	All	mixed	104/137	<50 vs. >147	2.67 (1.14–6.27)	age and sex	8
Torres-Durán et al. (2014) ^b^ [56]	Spain	HCC	M/F	>30	NS	192/329	<100 vs. ≥200	2.42 (1.45–4.06)	sex, age and environmental tobacco-smoke exposure	7
Duran et al. (2014) [57]	Spain	HCC	F	>30	NS	140/212	≤100 vs. ≥200	2.84 (1.58–5.09)	age and environmental tobacco exposure	7
Torres-Durán et al. (2015) ^a, b^ [58]	Spain	HCC	M/F	>30	NS	216/329	<200 vs. ≥200	2.11(1.43–3.11)	age and gender	7

^a^: histology subgroup analyses; ^b^: radon-prone areas; PCC: population-based case‒control study; HCC: hospital-based case‒control study; M: male; F: female; NS: nonsmokers; OR: odds ratio; CI: confidence interval.

**Table 2:** Summary risk estimates for residential radon exposure (highest versus non/lowest) and lung cancer.

Study	No. of Studies	OR (95% CI)	Heterogeneity Test	
			I^2^ (%)	*p*-Value
All studies	28	1.48 (1.26–1.73)	67.5	0.000
Histological subtype				
Adenocarcinoma	12	1.58 (1.31–1.91)	30.9	0.144
Small-cell carcinoma	11	2.03 (1.52–2.71)	0.0	0.445
Squamous cell carcinoma	10	1.43 (1.18–1.74)	0.0	0.473
Other histological types	10	1.54 (1.11–2.15)	34.8	0.129
Study population				
Europe	18	1.77 (1.54–2.03)	0.0	0.473
North America	7	1.09 (0.94–1.27)	34.0	0.168
Asia	3	0.93 (0.42–2.06)	72.8	0.025
Study design				
Population-based controls	18	1.28 (1.09–1.50)	56.5	0.002
Hospital-based controls	8	2.10 (1.69–2.60)	0.0	0.595
Hospital and population-based controls	2	1.70 (1.13–2.57)	0.0	1.000
Smoking status				
Smokers	8	14.80 (6.27–34.90)	80.0	0.000
Nonsmokers	12	1.38 (1.03–1.84)	65.3	0.001
Ex-smokers	3	2.21 (0.85–5.76)	52.9	0.120
Sex				
Female	7	1.38 (0.98–1.94)	63.7	0.011
Male and female	20	1.52 (1.26–1.84)	69.5	0.000
Duration of radon measurements (months)				
≥12	16	1.19 (1.03–1.38)	43.5	0.033
<12	12	2.02 (1.70–2.40)	0.0	0.699

OR: odds ratio; CI: confidence interval.

**Table 3:** Dose‒response effects of radon for lung cancer and histological types.

Study	Study Location	EOR per 100 Bq/m^3^ (95% CI) Radon Concentration				
		Overall	Adenocarcinoma	Small-Cell Carcinoma	Squamous Cell Carcinoma	Other Histological Types
Pershagen et al. (1994)	Sweden	0.10 (0.01–0.22)	0.17 (0.01–0.42)	-	-	-
Darby et al. (1998)	UK	0.12 (−0.05–0.33)	0.18 (−0.09–0.45)	0.20 (0.02–0.38)	−0.05 (−0.23–0.13)	0.03 (−0.19–0.25)
Kreienbrock et al. (2000)	Germany	0.13 (−0.12–0.46)	-	0.11 (−0.17–0.47)	-	-
Lagarde et al. (2001)	Sweden	0.28 (−0.05–1.05)	-	-	-	-
Wang et al. (2002)	China	0.19 (0.05–0.47)	-	-	-	-
Kreuzer et al. (2003)	Germany	0.08 (−0.03–0.20)	−0.02 (−0.23–0.22)	0.23 (0.02–0.47)	0.05 (−0.14–0.27)	-
Bochicchio et al. (2005)	Italy	0.14 (−0.11–0.46)	0.36 (−0.1–1.05)	0.22 (−0.21–0.89)	0.19 (−0.12–0.60)	−0.46 (−0.76–0.18)
Sandler et al. (2006)	USA	0.13 (−0.23–0.50)	0.20 (−0.19–0.59)	0.17 (−0.35–0.69)	−0.18 (−0.39–0.37)	0.21 (−0.35–0.78)
Wilcox et al. (2008)	USA	0.05 (−0.14–0.56)	-	-	-	-

EOR: excess odds ratio; CI: confidence interval.

## Supplementary Materials

The following are available online at https://www.mdpi.com/1660-4601/17/4/1457/s1, Figure S1: Funnel plots for the meta-analyses: overall pooled analysis, Figure S2: Funnel plots for the meta-analyses: histology subgroup analyses, Table S1: strategies of electronic database searches.
